# Introductory Address to the Class of Rush Medical College

**Published:** 1860-12

**Authors:** J. V. Z. Blaney


### INTRODUCTORY ADDRESS TO THE CLASS OF RUSH MEDICAL COLLEGE.

BY PROF. J. V. Z. BLANEY.

Gentlemen of the Medical Class:

This evening inaugurates the Eighteenth Annual Course of Lectures in Rush Medical College, and you are assembled to receive from us, your instructors, and we to offer to you a welcome to our institution. This welcome we now most cordially extend you, and while so doing, we cannot avoid congratulating ourselves and the friends of the Institution upon the fact, that the number of students in attendance upon the preliminary course just past, and present on this, the first day of the present session, is much greater than on any previous year since the foundation of the institution. This fact we cannot but accept as an earnest that our previous efforts, to afford to students of the Northwest a thorough course of medical instruction, has met the approval of the profession at large, as well as of those of you whose familiar faces have been before us in previous sessions. While feeling this to be a reward for the past, we are also stimulated to further endeavors to merit the approbation of those whose favorable opinion we desire to secure, as those most capable of judging our efforts.

Prepared somewhat to anticipate a large class at this session by previous correspondence, we have, as far as practicable, improved our means of demonstration, and made all possible arrangements to secure your comfort and convenience during the course of instruction just opening, and we confidently hope that these arrangements will contribute to your advancement in your studies, secure your uninterrupted health during the session, and meet your approval at the close.

· Selected by my colleages to offer to you some words of advice and counsel to direct your course of study, I select as the subject of a short address, "The elements of success and causes of failure in the character and habits of medical students." In the short time within which I must condense my remarks, it cannot be expected that I should give more than a faint outline of so copious a theme, bnt I hope to be able to exhibit a few of the dangerous reefs and shoals which but too often make shipwreck of the brighest hopes and most confident anticipations.

In the life of every man there is an epoch, which more than any other, determines for him the question of success or failure. To most men, probably, this arrives at the time when they make choice of the business or profession which it is their intention to pursue for the remnant of their allotted period of earthly existence. At this period you, gentlemen, have all arrived, as is proved by your presence here with the object of commencing the study of an honorable profession. The propriety of the selection you now make, and the resolutions now determined, will perhaps more than any other causes, influence your future success or failure. Does it not then become you to pause upon the threshold, and ponder seriously the responsibilities you are about to assume, and calmly and dispassionately look full in the face the difficulties and discouragements which are liable to beset your path at every step of advance? Were you merely about to enter upon the *study* of the medical profession, without intending to pursue it as your life-long avocation, the remarks I am about to offer would be needless. No profession offers in its prescribed course of study more allurements than that of medicine. It may truly be compared to a vast and beautiful field glorious in perpetual sunshine, and gemmed with myriads of flowers, both gorgeous to the eye and delicious to every sense, waiting only the appreciative mind to quaff its beauties and revel in its ever-varying delights. Were this all; were there no beyond; I should have no word of caution or warning to offer, but I would invite you to enter its glorious precincts and regale yourselves to the full, without fear of being cloyed with profusion, or disgusted with repletion. But beyond these delightful fields of study lie the rugged steeps and weary ascents of the stern realities of professional life, and through and beyond these you must pass to attain the laurel and the bay which is to reward a well spent life. To gain the goal, you will have to leap the slough of despairing confidence, shun the allurements of other and more agreeable paths to fame, and avoid the slush and mire of empirical systems, which, like the Dismal Swamp, conceal their fatal depths of ooze and mud with a thin skim of velvet moss, which seeming luxuriously soft and easy to the foot, but treacherously conceals the alligator and the adder, and once trusted with his footfall, permits the traveler to sink beyond recovery. These, and a thousand other difficulties, beset the path, and fortunate and brave is he who, at the end of a successful life, can look back and say with truth, I have overcome them all.

There is a trite saying, that "forewarned is forearmed." If this is true, would it not be a false and short-sighted generosity which would permit a young man to enter upon a journey beset with difficulties, which threaten to prevent its accomplishment, without informing him of its inevitable perils, preparing him to meet them with courage, and furnishing him with the weapons for his defence. Yet such would be the course of your instructors did they permit you to enter upon the study of your profession without sounding a single note of warning.

Suppose that you were about to undertake a journey in performance of a responsible mission, what inquiries should you make before you entered upon your duties? First, you would inquire into the nature of the mission, and the kind and amount of responsibility you would assume. Next, you would ask yourself whether or not you had the ability to perform the duties devolving upon you, and to answer this you would need to know the difficulties which would present themselves in the way of success. Lastly, you would wish to determine the course of conduct necessary to pursue in order skillfully to accomplish your purpose. Precisely such should be the course of self-examination pursued by each of you just entering upon a journey which is to last your whole life long, and upon a mission, than which few that a man can be called upon to perform, are more important, more arduous, or require higher qualities of mind or more mental and physical endurance.

Let us pursue this course of inquiry and examine—First: What is the nature, and what the amount of the responsibilities of the medical profession?

Gentlemen, if you could be made fully to appreciate the responsibilties you are to assume as medical men and yet feel willing to go forward in your course, you are brave men. You have before you no life of ease or gaiety, but one of severe mental application and physical labor, of constantly recurring trials of courage and trials of patience, of professional emulation and of non-professional criticism, of misconception, of reproach and of ingratitude, and with all this you have the perpetual burden of the responsibility of human life. Did you ever happen to be in a court of justice when the twelve men, to whom the law had submitted the decision of a charge of murder, came into court with their verdict of guilty stamped on their pale faces? Have you seen the prisoner at the bar, stained perhaps with numerous crimes, strain with haggard but eager gaze upon the foreman, from whose lips were about to issue the words, to him, of life or death? Have you seen that sea of human faces, pale and serious, riveted with breath restrained to catch the single word which dooms the wretched man to the scaffold, and yet failed to see, that each of those twelve men felt himself burdened with the responsibility of a human life, and that the burden was almost more than he could bear? And yet every day, nay, almost every hour of your life, as a practising physician, you will assume alone, not sharing with others, a responsibility equally great, and that of lives not justly forfeited by crime, but lives which, if preserved, might be of the highest use to our common humanity, lives of the pure and innocent, lives in which are bound up all the affections, of whole families, lives of the great and good.

Do you appreciate this burden you are about to assume? Then pause and ask yourselves if you are, or ever will be, prepared to take this yoke upon you. If not, then turn back while yet there is time and find your proper place in other paths of life. If yea, then, with God's help, acquit yourselves like men!

Do not presume that, by these remarks, I would discourage you from pursuing the profession of your choice. Not so. I only wish that you should fairly see and appreciate the worst, that you may gird yourselves the more strongly for the contest, and be fortified in your foreknowledge, and thus prepared in advance for the struggle which sooner or later must come. But these are not the sole responsibilities which will rest upon you as medical men. You have duties to the profession itself, to maintain its honorable character before the world as enlightened, liberal, benevolent and progressive. And as every individual is, in his own person, a representative of the profession at large, his character, his proficiency and his labors for its advancement, or his neglect thereof, all count in the estimate made by community of the profession itself. No man can accept of the rights, privileges, and position conferred upon him by his admission as a member, of any association, without tacitly acknowledging the claims of such association, that he will sustain its honor and contribute to its advancement. As this claim is enforced by no penalty, but is the result of reciprocal interchange of tacit pledges, it is the more strongly binding upon the honor of the individual, and can never be ignored without disgrace and loss of self-respect. It will not do to close your eyes to these claims and fold your arms in negligent contempt, for your own consciousness will perpetually urge them upon notice, and you will be self-condemned, as a useless drone, in the busy hive, peopled with your active and successful compeers.

With this brief sketch of your responsibilities, we pass on to the next inquiry—Have you the ability, the powers of mind, and the mental training requisite to the performance of these duties?

Know thyself is the most difficult of all knowledge, and yet one of the most essential elements of success. Many men live out their lifetime without ever learning their real capabilities, much less detecting their deficiencies. They may consequently be straining every nerve in a direction in which they never can succeed, while in other walks of life, demanding different qualities of mind, they might with half the effort distance their competitors. And when we reflect that the analysis of a mental organization, which should indicate the qualities fitting a man for any social position, is so difficult to be made by the oldest and wisest metaphysicians, it is no longer a wonder that frequent fatal errors should be made in the choice of a profession by the young and inexperienced. Again, as no two minds ever have or ever will exhibit the same combination of qualities and powers, so nothing more than the merest sketch, in outline, of the most prominent features, can be indicated to guide a young man in his selection.

In order to attain superior excellence in the Medical profession, there are a few prominent traits of mind which are so essential that I shall endeavor briefly to indicate them:

The medical man should have quick perceptions, and the faculty of comparison. Many physicians, for want of these powers, fail in diagnosis; the first duty of the physician, in every case to which he may be called. Men differ very much in this regard. Some medical men seem almost to have intuitive perceptions, which enable them to detect, and guide them almost unerringly in the analysis of symptoms. Such men will make a correct diagnosis while others have scarcely seen the outward signs, much less appreciated the train of thought by which they have arrived at their conclusions. The former have a fitness for the profession which the latter can never attain by the longest experience. It is only by the consciousness of the want of this faculty, which, it is true, is not acknowledged, but which would be a powerful incentive, that I can account for the fact that some men who have commenced their career in the right paths and under favorable auspices, have afterward chosen the easier paths of empiricism, where diagnosis is unnecessary, and all that is required is to prescribe for symptons as they arise, without any reference to pathological conditions or to the existence of causes to be removed. This must be a very easy and comfortable way to practice, but it appears to me must be rather unsatisfactory so far as a consciousness of filling any enviable position is concerned, for any old lady can take up the book, read off the symptoms of headache or toeache, and order the inevitable "nux, pullsatilla, camomilla, or mercurius," and dose out the required number of little sugar plums from the delicate little vial. It is all very plain sailing, and those who prefer it must be easily contented with such rivalry as I have just indicated. Indeed, I am at a loss to discover what is the use of the practioner at all where this is all there is to be done. But the worst rub of all must be the ever present conciousness of being a useless supernumerary in society, and of being paid for usurping the province of those elderly matrons who are always ready to give their services without fee.

Accuracy in diagnosis may, of course, be acquired by careful study of symptoms and by experience, but there is an immense difference in men in regard to the facility with which it is acquired, and with some there is a total inaptitude, which, when discovered, should be sufficient to deter him from further practice of the medical profession.

Decision of character and nerve are qualities which the medical man must possess in a high degree. These, if not innate, are seldom acquired, and the conscious want of them should at once deter any man from entering the arena of professional life. The want of these qualities is so evident to every one, so easy of detection by the patient and his friends, that a want of confidence and falling off of practice is the inevitable result, no matter how favorable the other conditions which ushered the young men into business.

Nothing sooner begets a want of confidence in the skill of a medical man than the appearance of wavering and hesitation. This is different from uncertainty in diagnosis, of which, if he is doubtful, he shows no want of decision or of nerve by confessing the fact and calling counsel. This, indeed, it is his duty to do, and sensible people, instead of losing confidence, will rather be impressed with reliance on his sincerity, and be more willing to trust him again in serious cases. Indeed, the refusal to call counsel, where the uncertainty of the physician is discovered, leads naturally to the supposition that he fears detection of some mistake, or that he is conscious of his own deficiencies and fears the exposure which an intelligent counsel would detect. Though I would deprecate the unnecessary calling of counsel in plain cases where nothing is to be gained, yet, where the anxiety of the patient or his family is evident and they appear to wish it, the attending physician should rather anticipate the wish of the family, provided a competent and respectable practitioner can be obtained. If any irregular man is proposed, respectfully decline to meet him, stating fairly your professional reasons for so doing, and if it be insisted upon, retire from the case rather than sacrifice your self-respect.

The want of decision to which I refer is rather the result of a want of self-confidence, a fickleness of purpose, and an indisposition to assume responsibility. Where this is the consequence merely of a consciousness of deficiency of acquirement in the principles of the profession, or a want of experience, it is remedied in time by study and practice or hospital instruction. But where it is a vice of mental organization it totally unfits a man for the active duties of the practice of medicine.

Nothing can be more deplorable than the condition of an undecided physician when he has to cope with an unusual form of disease, or has a succession of serious and difficult cases. As long as he has only ordinary ailments to manage, he gets on swimmingly, but let one of those fearful epidemics occur, where the best informed and bravest of the profession are at fault, those times that try men's souls, and where is he then? Sickened, desponding, faithless, in regard to the principles of his profession, he either falls himself a victim to the disease, or he seeks refuge for his craven soul in one of the easy chairs of empiricism.

That which I have termed *nerve* is no less essential than decision of character. It is, par excellence, one of the distinguishing characteristics of every eminent surgeon, and implies firmness of purpose, self-confidence, and confidence in the profession, fearlessness in assuming responsibility, great power of will, and perfect self-control, and, while the surgeon or obstetrical practitoner can only succeed when he possesses these qualities in strong development, a lack of them unfits a man for all branches of practice.

True, other qualities of mind, such as are essential to secure success in any undertaking, are equally necessary to the medical man, and however great, however grasping the intellect, it will find, in this department of study, full scope for its most exalted powers. But the qualities I have specially mentioned, I consider as absolutely essential to every man, in order that he may sustain even a respectable position as a practitioner.

Having pursued thus far your self-examination and determined to pursue the study of medicine as your profession, you should then determine the conduct on your part necessary to accomplish the study and most fully prepare you for its practical duties.

In this connection I can only hope to offer a few hints which may serve as finger posts on your journey, leaving minute details to your own good sense for their suggestion.

The first suggestion I offer is the necessity of *systematic* use of time. By this I mean the practice of setting apart a particular time of day for a particular duty, such an hour for a particular study, such an hour for another study, such an hour for exercise, or out-door employment. Believe me that by the adoption of this system you will accomplish immensely more in a given time than you could by attempting to study without any prescribed system. Of course this system will, at times, be unavoidably broken in upon by unusual occurrences, but if you are firmly determined to carry out your system, you will succeed, with a large proportion of your time, in working up to rule. Try it for a time, and my word for it, you will soon be surprised at the greater amount of reading you will accomplish. Nothing is more apt to grow upon students than a vicious habit of unprofitable lounging, either in each other's rooms, or in other lounging places. Were a record kept of the hours thus spent by many students, for a single year, it would scarcely be believed what an amount of valuable time had been lost. I do not, by any means, deprecate the occasional visiting each other for relaxation and rational conversation. Indeed, if the subjects of your studies form the basis of your talk, within certain limits, such a practice has its advantages, by eliciting each other's views, assisting each other's comprehension, and impressing facts and principles upon the mind by a familiar review. These visits should, however, only be indulged in during the hours devoted to relaxation, and respect should also be had to the hours of study of others, that the time may not be frittered away which it was their intention to devote to study.

*Make use of little moments of interval occurring unavoidably between your regular duties.* This is the secret by which some men accomplish so much more than others. Every moment, even an interval of five minutes while waiting an appointment, is devoted to reading, and in the aggregate of years a vast amount of knowledge is acquired. We are told that a certain authoress of note, who was lady in waiting to a queen, wrote several of her most valuable works in the few moments of each day that she was waiting the approach of her royal mistress. In the case of physicians and surgeons of large practice, these little intervals occurring between calls are the only times in which to accomplish all the reading necessary to keep pace with the advance of the profession, or write the notes of cases or other observations of value which make up their public reputation. This hint might be considered trivial if it applied merely to the loss of time during student life, but if we reflect that the habits then formed are apt to cling to a man his whole life long, it assumes an importance which may determine the question of mediocrity or superiority.

*Cultivate a habit of close attention in the lecture-room.* This is important in view of the fact that your lecturers, speaking for the most part extemporaneously, and giving to you the result of their experience, will often, under the inspiration of a moment of enthusiasm, throw out valuable suggestions which may prove of immense service to you in after life, but which will probably never go upon paper, and once lost from inattention is lost forever. Again, unless you become yourself a public teacher you can never know how much inspiration is given to a speaker by an attentive audience, or how difficult the effort to feel himself interested, and do justice to either the subject or himself, when a portion of his audience are listless, heedless, or perhaps attending to something else, while he is vainly laboring to drive knowledge into their resisting crania. The question is often asked by students whether it is of advantage to them to take notes during lecture time. The answer must be qualified. That the fact of taking notes induces a habit of undivided attention to the lecture there can be no doubt, and if the attempt is only made to set down the heads of the discourse, without trying to adopt the language of the lecturer, as a general rule the result is good. The notes themselves are valuable as a synopsis of the reading required to review the subject. The objection to taking notes during *demonstrative* lectures is this, that, while endeavoring to write down the remarks of the lecturer the demonstration may be lost, and of the two the latter is the more valuable. The distinction may then be made between a lecture which is purely didactic, and one which is mainly demonstrative; in the former the practice of taking notes, if dexterously done, so as not to lose one remark in setting down the other, is good, in the latter more is liable to be lost than gained by the practice.

The question may arise with some in regard to the relative amount of attention which it is desirable to pay to the several departments of study during each course of lectures. This query can only be answered with reference to the amount of previous reading enjoyed by each student. If you have commenced the study of medicine but a short time previous to your first course of lectures, it is desirable that you should pay more special attention to what might be termed the rudimentary branches, viz: Anatomy, Chemistry, Materia Medica and Physiology. Do not, however, neglect a regular attendance upon the more advanced and practical branches, for you will learn much from the demonstrations and teachings, that will render your reading during the interval between your courses of lectures much easier of comprehension and more satisfactory. Learning also the practical application of your more rudimentary branches, gives to them an interest which they would not otherwise possess. My advice then is, that while you attend and endeavor to learn all you can from all the courses of lectures, to which you have access, let the most of your time given to study outside of the lecture-room be spent in the dissecting-room, or in the study of the rudimentary branches, so that becoming thoroughly grounded in these you may be the better able fully to appreciate the practical branches in your second or third course.

If on the other hand you have had a pretty thorough course of previous reading, you will find it to your advantage to pay as much attention to one branch as another. Experience, I believe, has fully proven that attendance on one course of medical lectures upon each branch is wholly unsatisfactory. The first course gives to the student merely a vague outline view of the immense field he has to travel, without permitting even the quickest intellect to attain that complete and minute knowledge in detail, which is essential, in order that he may use his information to advantage in practice. Even the third course is of great advantage to the best read students, and the remark is not unfrequently made by gentlemen of the profession, who after a greater or less number of years of active practice have attended a full course of lectures in a good medical institution, that this last course was far more useful to them than those of their student period, not that the lectures were better or more complete, but that they themselves were more appreciative. Do not, gentlemen, allow yourselves to presume that any one of you makes an exception to this rule. You may have had an unusual amount of reading, you may be very quick of comprehension, you may have enjoyed unusual advantages in the office of your preceptor, but let not these advantages breed in your minds the self-conceit that you are an exception to the rule. Be not in haste to graduate. Thousands of practitioners now unable from the pressure of business to attend a medical school for a single session, would be willing to make almost any sacrifice to attain the knowledge they feel they have lost by not extending longer their study. With many a man it is keenly felt to have been to him a fatal mistake to have graduated too soon, and to have made the difference with him between remaining for life an ordinary practitioner in a small village, or becoming a leader in some special department of the profession.

The next suggestion I offer is, *to give as much attention as possible to clinical instruction.* I can scarcely hope in the short space into which I must crowd my remarks to be able fully impress upon you the importance of the facilities offered to you for becoming familiar with bedside practice. It can hardly be exaggeration to say that a single case, thoroughly explained and commented upon by a competent clinical instructor, advances a student further in diagnosis and treatment, than a hundred others seen in ordinary practice without an experienced guide to demonstrate its features. An experienced teacher, whether in the surgical or clinical wards of a hospital, will in the clinical lectures of a single session comprise in a nutshell the experience of a long life of practice, and that with a minuteness of detail and accuracy of demonstration which impresses the subject in a manner never to be forgotten. The well earned reputation of the men connected with prominent medical schools attracts to them the most serious and important cases which are only occasionally found in a limited business, but when occurring and well or ill treated go far to establish or depress the standing of every general practitioner.

In your clinical studies let me impress upon you the importance of acquiring a habit of minuteness in the examination of symptoms and in making comparison between the signs of healthy and diseased conditions of functions. First familiarize yourselves with the normal states of organs and the normal performance of functions, by examining the pulse, tongue, breathing, sounds of the lungs and heart, etc., of each other, or of those presumed to be in good health, then in the hospital wards endeavor to ascertain the aberrations in the symptoms or physical signs which mark the presence of disease. Never feel satisfied with the dictation of your preceptor that things are thus and so, but try and perceive for yourself and record upon your memory the signs or symptoms with all their normal and abnormal associations, comparisons, and indications. Then, when you have returned from your clinical lecture make copious notes of the lecture and of your own observations. If you have access to a library, study out in detail all you can find bearing directly or remotely upon the case in hand. In your notes make references to all the authorities upon the subject which you can find, and, as often as may be, make out a full report of the case as if for publication. Submit your notes freely to the criticism of those who have more experience than yourself in observation and in writing, and are willing to correct them, and thus you will gain the habit of making up notes of cases, and obtain a facility of composition and correctness of expression which will be invaluable to you in after life, and go far to assist in effecting your reputation with the profession at large.

There is many a physician whose extensive practice and unusual experience would, if published, make for him a high reputation at home and abroad, who remains in obscurity, and whose results are lost to science, in consequence of his want of facility in writing and his indisposition to commit his ideas to paper.

Another hint—*Never lose the opportunity to be present at and assist in a post-mortem examination.* No one of the sources of information is more neglected by the profession at large than this, the only means of attaining exact knowledge of pathological conditions, and of confirming or disproving the significance of physical signs and symptoms of disease. Investigation in this mode is almost exclusively confined to the physicians and surgeons of hospitals, or the teachers in schools of medicine, when invaluable opportunities are constantly occurring, in private practice, which, if properly improved, would do much to increase our knowledge and advance the reputation of the fortunate investigator. One reason for this neglect, I cannot but think, is due to the fact that comparatively few general practitioners know how properly to conduct a post-mortem examination; or, if made, are able to place a proper comparative estimate upon the indications of disease or abrasions from the healthful appearance of organs. It is during your dissections that you are to take your first lessons in this direction. Let no opportunity pass to observe and record upon the tablet of memory, the comparative appearances of diseased and healthy structures, and to become familiar with the symptoms corresponding to the cadaveric appearances. Every advance in a direction not common to the mass of your compeers, gives you an advantage over them in the struggle for position, and widens the distance between you and the empiric, by whom all such exact and critical knowledge, and all scientific inductions thence derived, are not merely neglected, but derided.

But, gentlemen, I will not longer detain you with advice, which is apt, like legislation, to fail of its effect if there is too much of it. I will only detain you, in conclusion, to inquire as to the objects you propose and the aims you have set before you to attain in your selection of the medical profession. If wealth be your object—if you have taken up the study of the profession *merely as a business* by which you expect to attain a fortune in a short period—I fear you have made an egregious mistake. No profession is in general so badly paid for the amount of study required for its attainment, for the mental toil and responsibility incurred, or the physical labor and exposure entailed in its pursuit. The number of its votaries which ever make more than a respectable competency as the legitimate results of their practice is small. The mass of physicians, who have ever become wealthy, have made their fortunes by other modes. Only those who attain the summit of fame and ambition, as the leading surgeons or physicians of large and wealthy cities, can boast of wealth fairly attained in the practice of the profession.

Obviously then to attain this, if it alone be your object, the only sure and direct path is that which will most surely lead to the highest eminence as a professional man. It is, however, to be hoped that a large proportion, if not all of you, are influenced by higher motives; that for you, the wholesale charge, so often made, is false, "that in America the 'Almighty Dollar' is the only deity that is sincerely worshipped;" that you have embraced the medical profession as one which will give scope to the cultivation of the highest intellectual faculties and to the most enlarged benevolence, and which may lead to an enviable position in society, to be attained by merit and by merit alone. If these be the sentiments which animate you, if you work and toil unceasingly with enlarged minds and ardent impulses after the true and the good, not turned aside by the lures and charms of political ambition, or rapidly acquired wealth, there is hope that you may attain all and more than all that you seek or anticipate.

> "So live, that when the summons comes to join
> The innumerable caravan, that moves
> To that mysterious realm, where each shall take
> His chambers in the silent halls of death,
> Thou go not like the quarry-slave at night,
> Scourged to his dungeon; but sustained and soothed
> By an unfaltering trust, approach thy grave
> Like one who wraps the drapery of his couch
> About him, and lies down to pleasant dreams."
>
> —*Bryant's Thanatopsis.*

